# A Rapid Consolidation Route for Recycled NdFeB Powders and the Role of Particle Shape in Grain Growth

**DOI:** 10.3390/ma18215029

**Published:** 2025-11-04

**Authors:** Fabian Burkhardt, Alba Berja, Laura Grau, Matija Kreča, Lindrit Krasniqi, Benjamin Podmiljšak, Kristina Žužek, Carlo Burkhardt, Spomenka Kobe, Adrián Quesada, Tomaž Tomše

**Affiliations:** 1 Department for Nanostructured Materials, Jožef Stefan Institute, Jamova Cesta 39, 1000 Ljubljana, Slovenialindrit.krasniqi@ijs.si (L.K.); benjamin.podmiljsak@ijs.si (B.P.); tina.zuzek@ijs.si (K.Ž.); spomenka.kobe@ijs.si (S.K.); tomaz.tomse@ijs.si (T.T.); 2 Instituto de Cerámica y Vidrio (ICV), Consejo Superior de Investigaciones Científicas (CSIC), Calle Kelsen 5, 28049 Madrid, Spain; a.quesada@icv.csic.es; 3 Departmento de Física de Materiales, Facultad de Físicas, Universidad Complutense de Madrid, 28040 Madrid, Spain; 4 Institute for Precious and Technology Metals (STI), Pforzheim University, Tiefenbronner Str. 65, 75175 Pforzheim, Germany; laura.grau@hs-pforzheim.de (L.G.); carlo.burkhardt@hs-pforzheim.de (C.B.); 5 Jožef Stefan International Postgraduate School, Jamova Cesta 39, 1000 Ljubljana, Slovenia

**Keywords:** Radiation-Assisted Sintering (RAS), NdFeB permanent magnets, magnet recycling, critical raw materials, sustainability

## Abstract

The recycling of NdFeB magnets is essential to reduce reliance on critical rare earth elements and mitigate the environmental burden of virgin magnet production. Hydrogen Processing of Magnetic Scrap (HPMS) offers an efficient method to extract magnet powders from end-of-life (EOL) products, yet oxidation and microstructural degradation during powder preparation limit the magnetic performance of recycled magnets. In this work, rapid Radiation-Assisted Sintering (RAS) was systematically evaluated for the first time as a consolidation route for HPMS-derived powders. Magnets prepared via RAS exhibited performance comparable to those obtained by conventional sintering. When oxygen uptake during milling was prevented, the addition of 1 wt.% NdH_3_ to the already oxygen-burdened recycled powder improved the intrinsic coercivity and squareness of the demagnetization curve. The best-performing samples achieved B_r_ = 1.18 T, (BH)_max_ = 263 kJ/m^3^, and H_ci_ = 742 kA/m at 100 °C, surpassing the properties of the original EOL magnets. Furthermore, the study revealed that, when the HPMS powder fragments preferentially break along grain boundaries, the resulting near-equilibrium powder particles exhibit limited growth, thereby restraining grain coarsening. These findings highlight the strong potential of RAS for more energy-efficient magnet-to-magnet recycling and provide new insight into optimizing HPMS powder processing to achieve enhanced magnetic performance.

## 1. Introduction

Permanent magnets based on neodymium–iron–boron (NdFeB) alloys are indispensable components in energy-efficient technologies, including electric vehicles, wind turbines, and a wide array of electronic devices [1,2,3,4]. Their superior magnetic performance makes them critical for achieving sustainability targets in energy conversion and storage. However, the production of NdFeB magnets is heavily dependent on rare –earth elements (REEs), which are subject to significant supply risks [5,6]. More than 85% of the global REE supply is currently sourced from China, often through energy- and chemically intensive processing routes with notable environmental and geopolitical implications [7]. In response to these vulnerabilities, the European Union has formally listed REEs as critical raw materials and introduced the 2024 Critical Raw Materials Act [5], emphasizing the importance of recycling and circular material strategies for these strategic elements.

Recycling of NdFeB magnets, particularly through closed-loop magnet-to-magnet routes, represents a promising solution to reduce both environmental impact and supply chain dependency. Recent life cycle assessments have shown that magnet-to-magnet recycling, i.e., crushing end-of-life (EOL) magnets into powders, followed by re-sintering, can reduce energy consumption by more than 45% compared to virgin magnet production [8]. Among the emerging recycling methods, Hydrogen Processing of Magnetic Scrap (HPMS), which was developed by the University of Birmingham [8,9,10,11], has proven to be an efficient and selective approach for extracting magnet powders from EOL products without extensive mechanical or chemical processing [11]. However, achieving magnetic properties in recycled magnets equivalent to virgin magnets remains a central challenge, particularly due to oxidation and suboptimal microstructures of HPMS-type powders, and limitations associated with traditional sintering processes. EOL magnets typically exhibit higher oxygen contents, ranging from 2000 to 5000 ppm, in contrast to the 300 to 400 ppm observed in newly cast magnets [12]. This increased oxygen content leads to the formation of high-melting-point oxides [12] in the RE-rich phase, primarily located at the grain boundaries [13]. These oxides subsequently inhibit the necessary melting of the RE-rich phase and therefore proper densification during sintering [12,14], resulting in severely degraded intrinsic coercivity [13].

Conventional sintering, i.e., the traditional method for consolidating magnet powders, is energy-intensive and time-consuming [12]. It also carries a high risk of grain coarsening and phase degradation, especially when applied to recycled powders with pre-existing structural or chemical inhomogeneities [13]. These challenges hinder the full realization of HPMS as a green scalable recycling pathway.

Radiation-Assisted Sintering (RAS) [12] is a novel rapid consolidation technique with strong potential to address these limitations. By employing intense thermal radiation to enable high heating rates and short dwell times, RAS minimizes processing time while achieving full densification. Recent work by Tomše et al. [12] demonstrated that RAS can produce fully dense NdFeB magnets from standard micron-sized powders within minutes, offering energy efficiency at least tenfold greater than slow conventional sintering methods at the lab scale [12,14].

To date, RAS has not been systematically evaluated for its applicability to recycled NdFeB powders. In this study, we examined the influence of RAS-specific conditions on the magnetic performance and microstructural evolution of magnets prepared from jet-milled HPMS powders. Both RAS and conventional sintering fully restored the performance of the original EOL magnets when oxygen contamination in the recycled material was compensated by adding 1 wt.% NdH_3_ and additional oxygen uptake during milling was avoided. Furthermore, the investigation showed that, when coarse HPMS material is fragmented preferentially by intergranular cracking, i.e., along grain boundaries, grain growth during re-sintering is suppressed, yielding a grain growth factor of approximately 1.2.

## 2. Materials and Methods

### 2.1. Powder Feedstocks and Green Compact Preparation

Wind turbine end-of-life (EOL) NdFeB permanent magnets were sourced through STENA Recycling, Gothenburg, Sweden. They were processed via Hydrogen Processing of Magnetic Scrap (HPMS) [9] in pure hydrogen atmosphere at 3 bar pressure. The reaction was terminated when no more drop in hydrogen pressure due to absorption was observed. Two powder feedstocks with reduced particle size were prepared from the same EOL material by milling the coarse HPMS material using two different jet mills.

To prepare the first powder feedstock (*JM_3X_*), the HPMS powder was milled using a laboratory-scale MC DecJet^®^ 50 jet mill (Dec Group Dietrich Engineering Consultants, Ecublens, Switzerland). The DecJet^®^ 50 jet mill was placed inside an argon-atmosphere glovebox. The HPMS powder was not degassed before jet milling to preserve its brittleness. Milling conditions in argon processing gas employed venturi and grinding ring pressures of 7 and 5 bar, respectively. Initially, the material underwent several milling cycles (*JM_1X_* = 1 milling cycle, *JM_2X_* = 2 milling cycles, etc.). However, the final powder was prepared with three milling cycles as no further particle size reduction was achieved with additional milling.

The second powder feedstock (*JM_Industrial_*) was produced using an industrial-scale spiral jet mill (LabPilot M-Jet 10 with an M Class 5 classifier, NETZSCH GmbH, Selb, Germany). The HPMS powder was milled in nitrogen gas under 6.8 bar of pressure. A classifier was used to control the final particle size. The classifier speed was set to 5000 rpm.

The powders were magnetically aligned under a 6 T pulse field and compacted into cylindrical green compacts, with a diameter of ~17 mm and a height of ~15 mm, using cold isostatic pressing (CIP RP 2000 QC/LC, Recherches & Réalisations REMY SAS, Montauban, France) in a silicon mold contained in a vacuum/argon-sealed bag at 800 MPa. Selected samples were prepared with the addition of 1 wt.% of jet-milled NdH_3_ (D_v50_ = 3.3 µm). For powder blends, the milled NdFeB and NdH_3_ powders were mixed with mortar and pestle inside an argon glovebox until fully homogenized.

### 2.2. Conventional Sintering (CS), Radiation-Assisted Sintering (RAS), and Annealing

Fully dense sintered samples were prepared from the NdFeB powders and NdFeB/NdH_3_ powder blends using either conventional vacuum sintering (CS) or Radiation-Assisted Sintering (RAS) followed by post-sinter annealing. In all cases, the green compacts were loaded into the sintering furnace inertly in argon gas, and the systems were flushed three times with additional argon to ensure the complete removal of any oxygen trapped in the system.

CS was performed in a custom-built high vacuum furnace at 1∙10^−5^ mbar pressure using single-stage heating at 3 °C/min to 1100 °C with a dwell time (t_DWELL_) of 2 h. For CS, the samples were placed in an alumina crucible (boat-shaped) to ensure that they were located at the center of the heating zone.

RAS employed a modified spark-plasma sintering furnace (SPS-632LxEx, Dr. SINTER SPS Syntex Inc., Kawasaki, Japan) with dynamic vacuum conditions. A schematic illustration of the setup can be found in [12]. The green compacts were placed in a cylindrical graphite crucible electrically insulated at the bottom by graphite felt (Sigatherm GFA5, SGL Carbon GmbH, Wiesbaden, Germany) coated with boron nitride. The crucible dimensions were 38.4 mm inner diameter and 23.4 mm height, with a wall thickness of 4.2 mm. Under such noncontact conditions, the electric current flows through the crucible, which heats up and acts as a heat source, i.e., radiator. The heating was current-controlled from room temperature to 570 °C, and temperature-controlled from 570 °C to the sintering temperature (T_SINT_) of 1100 °C using a pyrometer. For the first powder feedstock (*JM_3X_*), the heating rate was 25 °C/min, and the dwell time (t_DWELL_) at T_SINT_ was 5 min, following the procedure published previously [12]. For the second powder feedstock (*JM_Industrial_*), t_DWELL_ was increased to 30 min to achieve full density due to higher oxygen content than in the JM_3X_ powder. The NdFeB powders contained interstitial and bonded hydrogen from the HPMS process, which was progressively desorbed during the heating stage up to ~600 °C [15]. Chamber pressures during the heating ranged initially from approximately 0.03 mbar to 1 mbar on average with a maximum of 7 mbar (desorption of hydrogen). After sintering, natural cooling under vacuum occurred.

Single-stage post-sinter annealing was carried out for all samples in the custom-built vacuum furnace, heating samples at 5 °C/min to 520 °C (T_ANNEAL_) with a t_DWELL_ of 2 h, followed by natural cooling.

### 2.3. Characterization of Powders and Bulk Samples

Particle size distributions of the NdFeB jet-milled powders and NdH_3_ powder were measured using laser diffraction (HELOS/BR, Sympatec GmbH, Clausthal-Zellerfeld, Germany) with R1 (0.18–35 µm) and R3 (0.9–175 µm) lenses. All powders were oxidized before measurement and dispersed using the ASPIROS dry disperser with an applied pressure of 1 bar of compressed air. The results were analyzed using the Fraunhofer (FREE) method.

Chemical analysis of the EOL magnet was conducted via inductively coupled plasma optical emission spectroscopy (ICP–OES, iCAP 7400, Thermo Fisher Scientific, Waltham, MA, USA). ICP samples were digested in aqua regia containing 5% nitric acid using microwave-assisted digestion (MARS6, CEM Corporation, Matthews, NC, USA) at 200 °C, diluted, and analyzed against a custom-prepared tuning solution.

Magnetic properties of the EOL magnet and sintered samples were measured using a permeameter (EP2 Permagraph, Magnetphysik Dr. Steingroever GmbH, Cologne, Germany), with heated coils allowing for measurements up to 250 °C. Owing to the measurement apparatus and the high coercivity of the samples, demagnetization curves were obtained at 100 °C to achieve full demagnetization. The samples were heated by the coils of the permeameter, and, once the coils reached 100 °C, the samples were held for 5 min at this temperature to ensure that, due to the thermal lag, the sample’s core could reach the desired temperature. This temperature is also representative of the typical operating conditions of permanent magnet-based electric motors, providing insights into the actual performance of the recycled materials.

Bulk density was measured using Archimedes’ principle with silicon oil infiltration (Densitec, Exelia AG, Zürich, Switzerland). Oxygen contents were determined using the LECO ONH836 (LECO Corporation, St. Joseph, MI, USA) via hot gas extraction, infrared detection of CO, and calibration against certified reference materials.

Microstructural analysis utilized scanning electron microscope (FEG-SEM, JEOL JSM 7600F, JEOL Ltd., Tokyo, Japan) combined with energy-dispersive X-ray spectroscopy (INCA 350 EDS, Oxford Instruments, Abingdon, UK). Grain size analysis was performed on four bulk samples: an EOL reference magnet, two samples composed of *JM_3X_* jet-milled powder using RAS and CS, and a sample prepared from *JM_Industrial_* powder using RAS. All sintered samples, except the EOL reference, contained 1 wt.% of NdH_3_. For each sample, three SEM micrographs were acquired at a magnification of 1000x at different positions of each polished and etched sample. The microstructures were etched for 2 min using a mixture of Cyphos 101 IL and HCl. In the SEM micrographs, the grains were manually traced (digitally) to create a binary outline mask, and the average grain diameter was determined using Feret’s diameter in ImageJ (Version 1.54g) [16,17], following the methodology outlined in [18].

## 3. Results and Discussion

### 3.1. EOL Magnet Characterization

**Figure 1:**
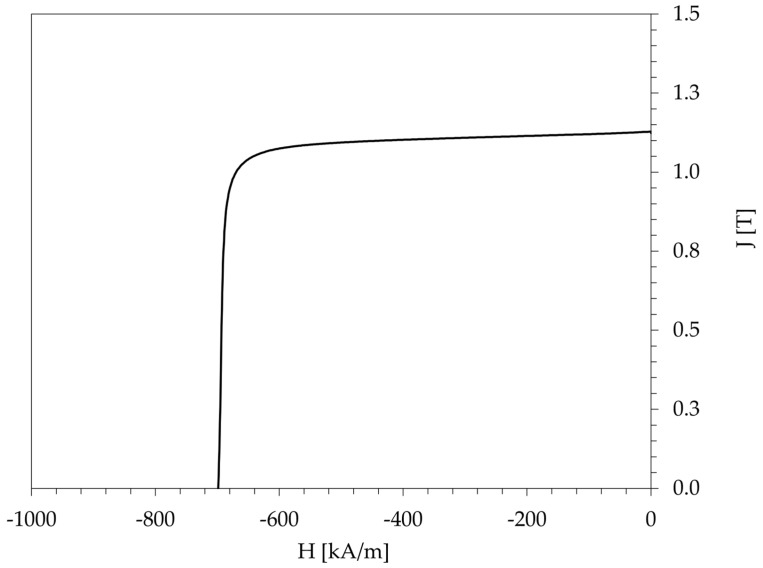
Demagnetization curve (measured with a permeameter at 100 °C) for the end-of-life (EOL) magnet.

The EOL magnet was characterized before recycling to establish the baseline properties at the end of the life cycle. Its chemical composition, density, and key magnetic properties are compiled in Table 1. Chemical analysis using ICP–OES revealed a dysprosium content of approximately 4.2 wt.%, among other alloying elements. At 100 °C, the magnet exhibited a remanence (B_r_) of 1.13 T, an intrinsic coercivity (H_ci_) of 699 kA/m, and a maximum energy product ((BH)_max_) of 241 kJ/m^3^. The demagnetization curve obtained at 100 °C has a squareness factor, defined as H_k_/H_ci_, where H_k_ is the knee point field at 0.9 B_r_ and H_ci_ is the intrinsic coercivity of 0.95. The curve is shown in Figure 1.

### 3.2. NdFeB Powder Feedstock Characterization

**Figure 2:**
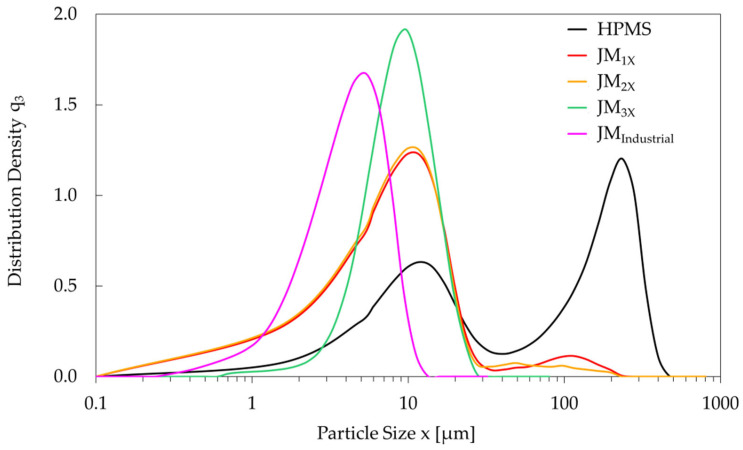
Particle size distribution density (q_3_) curves for the *HPMS* powder (black) and consecutive milling stages (*JM_1X_*—red, *JM_2X_*—orange, and *JM_3X_*—green) for the laboratory-scale MC DecJet^®^ 50 jet mill, showing how repeated milling narrows the particle size range. A curve for a powder milled on an industrial-scale jet mill (*JM_Industrial_*—magenta) reveals a smaller average particle size.

The oxygen content and particle size distribution for the HPMS and milled powders are listed in Table 2. The HPMS powder had an oxygen content of 0.47 wt.% and a D_v50_ value of 71.9 µm. Three consecutive milling steps using a laboratory-scale MC DecJet^®^ 50 jet mill (powder *JM_3X_*) reduced the D_V50_ to 8.9 µm. This value is slightly higher than the D_V50_ of the twice-milled powder *JM_2X_* (8.0 µm), which is attributed to the increased D_v10_ value of the *JM_3X_* powder due to the removal of a fraction of small particles during the third milling cycle, which are filtered out in the cyclone separator of the jet mill. Moreover, the D_v90/10_ ratio decreased from 9.6 (*JM_1X_*) to 9.0 (*JM_2X_*) and finally 3.6 (*JM_3X_*), revealing a narrow size distribution in the thrice-milled powder. This is also seen in the particle size distribution density curves shown in Figure 2. After the third jet-milling cycle (*JM_3X_*), the curve is significantly narrower due to the disappearance of the secondary peaks observed for powders *JM_1X_* and *JM_2X_* (reduction in D_v90_) and the removal of small particles (increase in D_v10_). The oxygen content initially increased from 0.47 wt.% (HPMS) to 0.56 wt.% (*JM_1X_* powder) and later decreased to 0.42 wt.% for the final *JM_3X_* powder. This reduction is in agreement with the removal of small particles of oxidized RE-rich phase, which is a common occurrence in inert atmosphere jet milling [19].

The *JM_Industrial_* powder, prepared using an industrial spiral jet mill LabPilot M-Jet 10, had a D_v50_ value of 4.3 µm (see Table 2), approximately half that of the *JM_3X_* powder. This particle size reduction is also evident in Figure 2, where the particle size distribution density curve corresponding to *JM_Industrial_* powder is shifted to the left relative to the *JM_3X_* powder. It is attributed to the difference in particle-on-particle collision impact during milling, evidencing superior efficiency of the industrial jet mill. Nevertheless, both powders have narrow size distributions with nearly identical D_v90/10_ ratios (3.6 and 3.5 for *JM_3X_* and *JM_Industrial_*, respectively; see Table 2), necessary to ensure a homogeneous microstructure in bulk sintered magnets.

The oxygen content in powders *JM_3X_* and *JM_Industrial_* was significantly different. While a reduction in the oxygen content for the *JM_3X_* powder confirmed that jet milling inside an argon glovebox prevented additional oxygen uptake, the industrial jet mill did not offer the same protection against oxidation as the oxygen content increased to 0.59 wt.% upon milling. Considering its more favorable oxygen level, powder *JM_3X_* was used as a feedstock material for further sintering trials (Section 3.3).

The oxygen content of the *JM_Industrial_* powder was found to be too high to achieve satisfactory magnetic performance in new magnets after re-sintering, and the material was used only to study the effect of particle size and shape on the grain-growth dynamics (Section 3.4).

**Figure 3:**
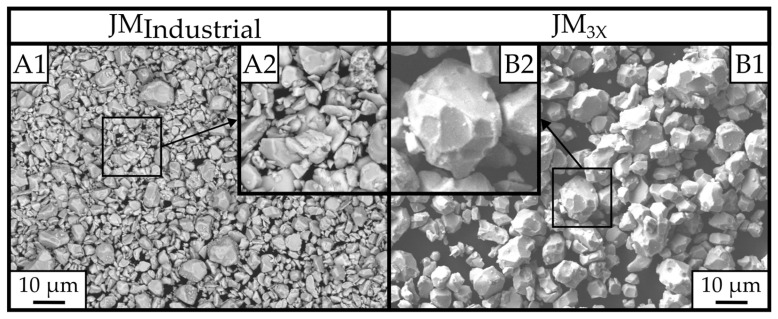
Powder morphology images by SEM microscopy at a magnification of 1000x. (**A1**) represents the finely milled *JM_Industrial_* powder, and (**B1**) represents the *JM_3X_* laboratory-scale jet-milled powder. (**A2**) shows a magnified section, highlighting the fractured shape morphology of the powder, compared to the faceted and more rounded morphology of the larger *JM_3X_* powder shown in (**B2**).

In Figure 3, the powder morphologies of the jet-milled powders are shown. The *JM_Industrial_* powder morphology (Figure 3(A1)) is fine and contains fractured particles, typical for high-impact jet milling. Some larger particles on the order of ~10 µm appear to be more rounded and similar in size to the *JM_3X_* powder. In the laboratory-scale *JM_3X_* powder (Figure 3(B1)), on the other hand, the majority of grains show a rounded and faceted morphology, with particles significantly larger than in the *JM_Industrial_* powder. Figure 3(B2) particularly highlights the faceted morphology of the particles.

### 3.3. Performance of Bulk Samples Prepared from JM_3X_ Powder

Bulk samples were prepared from the *JM_3X_* jet-milled powder by Radiation-Assisted Sintering (RAS) and conventional sintering (CS), followed by post-sinter annealing. Due to the increased oxygen content of 0.42 wt.% in the powder feedstock compared to conventional NdFeB materials at <0.30 wt.% [20], two samples were prepared with the addition of 1 wt.% NdH_3_ to compensate for the loss of liquid phase.

**Figure 4:**
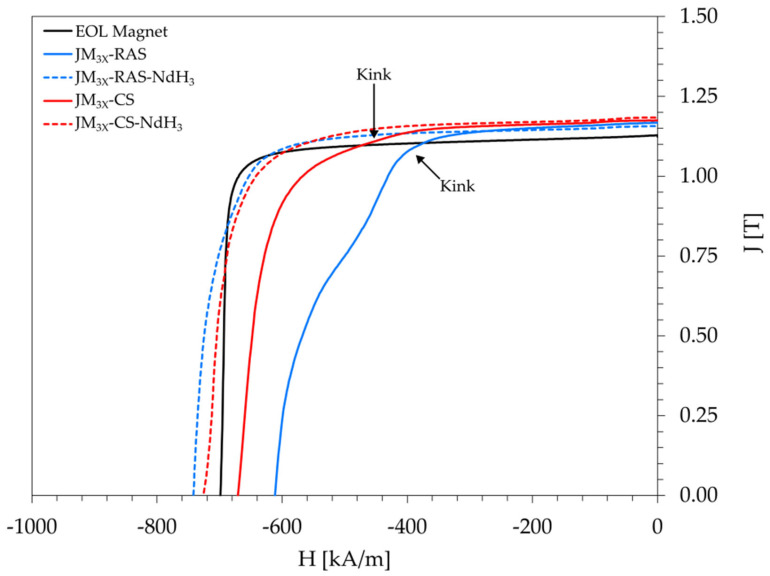
Demagnetization curves (measured at 100 °C) for bulk samples prepared from the *JM_3X_* powder by RAS or CS, followed by post-sinter annealing: EOL reference (solid black), *JM_3X_*-RAS sample without additive (solid blue), *JM_3X_*-RAS-NdH_3_ sample prepared with 1 wt.% NdH_3_ (dashed blue), *JM_3X_*-CS sample without additive (solid red), and *JM_3X_*-RAS-NdH_3_ sample prepared with 1 wt.% NdH_3_ (dashed red).

The magnetic properties, obtained at 100 °C, are listed in Table 3, and the correlating demagnetization curves for all the samples are shown in Figure 4. The measured densities of the samples prepared by RAS and CS range between 7.58 and 7.64 g/cm^3^, and the samples are considered fully dense.

For the *JM_3X_-RAS* sample prepared without the additive, the remanence (B_r_) reached 1.17 T at 100 °C, slightly exceeding the EOL magnet’s value of 1.13 T. However, the squareness factor of its demagnetization curve, H_k_/H_ci_, was only 0.68. The corresponding curve (Figure 4, solid blue curve) displayed a notable kink and was significantly less squared compared to the EOL magnet (solid black curve), with a squareness ratio of 0.95. The remanence of a conventionally sintered sample *JM_3X_-CS* prepared without the additive was 1.17 T, i.e., identical to RAS sample *JM_3X_-RAS*. On the other hand, the kink in its demagnetization curve (solid red curve) is notably less pronounced than the kink observed for sample *JM_3X_-RAS*. In turn, the squareness factor was improved from 0.68 (*JM_3X_-RAS*) to 0.78 (*JM_3X_-CS*).

For sample *JM_3X_-RAS-NdH_3_*, the addition of 1 wt.% NdH_3_ improved the curve’s shape (dashed blue curve) and increased its squareness factor to 0.86. Moreover, while the remanence remained almost unchanged at 1.16 T, the coercivity increased from 611 kA/m (no addition) to 742 kA/m. For sample *JM_3X_-CS-NdH_3_*, the NdH_3_ addition increased the coercivity from 671 kA/m (no addition) to 726 kA/m without reducing the remanence. In summary, the magnetic properties of samples prepared by RAS or CS with the addition of NdH_3_ are comparable. Their B_r_ values (1.16 and 1.18 T), H_ci_ values (742 and 726 kA/m), and (BH)_max_ values (254 and 263 kJ/m^3^) exceeded the properties of the initial EOL magnet by ≈2–4% (B_r_), ≈3–6% (H_ci_), and ≈5–9% ((BH)_max_). These results confirm that the RAS process is a viable alternative to conventional sintering in the short-loop recycling strategies for end-of-life NdFeB magnets, ensuring comparable magnetic performance while significantly reducing sintering time.

**Figure 5:**
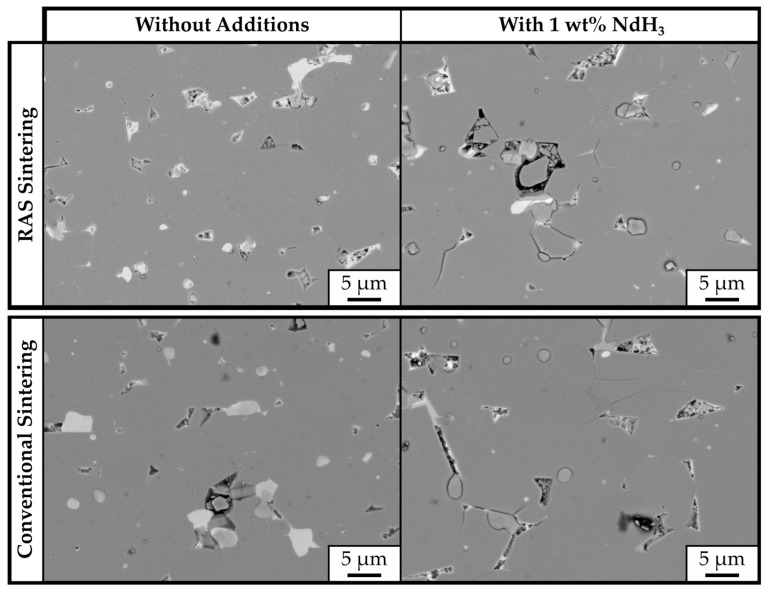
SEM micrographs of bulk samples prepared from powder *JM_3X_*. Top left: RAS sample without additive (*JM_3X_-RAS*); top right: RAS sample prepared with 1 wt.% NdH_3_ (*JM_3X_-RAS-NdH_3_*); bottom left: CS sample without additive (*JM_3X_-CS*); and bottom right: CS sample prepared with 1 wt.% NdH_3_ (*JM_3X_-CS-NdH_3_*).

**Figure 6:**
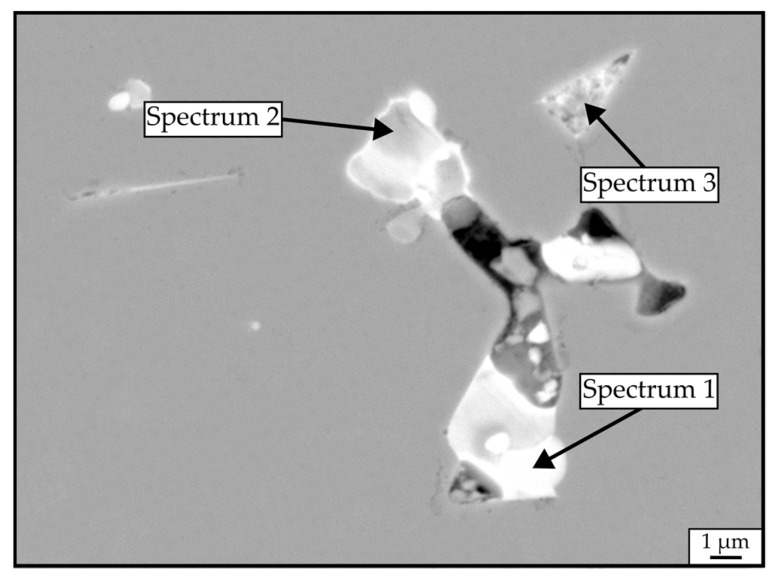
Representative SEM micrograph in BSE mode of a *JM_3X_-RAS* sample prepared without the addition of NdH_3_. Compositions of the bright Nd-rich phases were quantified by EDS, and the results are displayed in Table 4.

The backscattered-electron (BSE) SEM images, taken on the polished cross-sections of RAS and CS samples, are shown in Figure 5. The samples’ microstructures are comparable, consisting of a gray Nd_2_Fe_14_B matrix phase and brighter RE-rich phases located in the triple pockets. Figure 6 shows a higher-magnification BSE-SEM image of sample *JM_3X_-RAS* with marked secondary phases analyzed with EDS. Their compositions are shown in Table 4. All three analyzed areas contain significant amounts of oxygen, ranging between 54.5 and 63.3 at.%, showing compositions close to Nd_2_O_3_.

The origin of the kink in the demagnetization curves of samples *JM_3X_-RAS* and *JM_3X_-CS* is attributed to the presence of oxides. Vasilenko et al. [21] reported that oxides, such as NdO_x_ and Nd_2_O_3_, impede the development of continuous thin layers of the Nd-rich phase along the grain boundaries, leading to incomplete magnetic insulation of the Nd_2_Fe_14_B grains. This hinders coercivity development by reducing the effective exchange decoupling between the Nd_2_Fe_14_B grains. Moreover, poor grain-boundary wetting and oxide grains create weak spots in the microstructure, with a locally reduced nucleation barrier for reversal, leading to a multi-step reversal process [21].

What distinguishes rapid RAS from slow conventional sintering is the duration of the sintering cycle, i.e., the heating rate and dwell time at the sintering temperature of 1100 °C. The heating rates for RAS and CS were 25 and 3 °C/min, and the dwell times were 5 and 120 min, respectively. In RAS, the time during which liquid-phase sintering occurs may be too short to facilitate homogeneous redistribution of the liquid RE-rich phase along the grain boundaries, leaving grains with incomplete magnetic insulation that act as reverse-domain nucleation sites. Consequently, the kink in the curve was more pronounced for the RAS sample.

The positive effect of NdH_3_ on the magnetic properties, i.e., the coercivity increase and kink elimination, is attributed to an increased volume fraction of the liquid phase during sintering, leading to better magnetic insulation of hard-magnetic grains with the RE-rich grain-boundary phase in the final samples. For the *JM_3X_* powder, which has a moderate oxygen content of 0.42 wt.%, the addition of 1 wt.% NdH_3_ was sufficient to compensate for the absence of a metallic Nd-rich phase in the recycled NdFeB material caused by oxidation. At the same time, this amount was low enough to avoid a reduction in remanence, which might not be achievable with HPMS-based powders with higher oxygen content.

### 3.4. Effect of Particle Shape on Grain Growth

**Figure 7:**
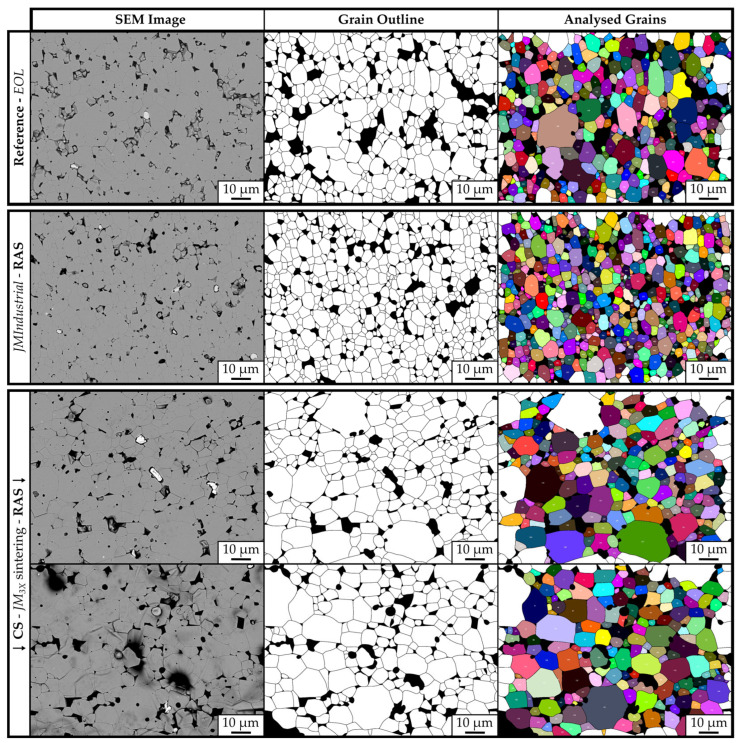
Representative SEM images (1000x magnification) of polished and etched cross-sections of four samples. Top row: EOL magnet. Middle row: RAS sample prepared from *JM_Industrial_* powder with the addition of 1 wt.% NdH_3_ (*JM_Industrial_-RAS-NdH_3_*). Bottom row: RAS and CS samples prepared with 1 wt.% NdH_3_ (*JM_3X_-RAS-NdH_3_* and *JM_3X_-CS-NdH_3_*). Left column: etched microstructures showing grain morphology. Middle column: binary outline masks with grain boundaries traced (grains in white; pores and secondary phases in black). Right column: ImageJ overlay maps with individual grains colored and numbered. Only fully enclosed grains were included in the analysis.

To investigate the grain-growth dynamics for samples prepared with RAS or CS, the polished cross-sections of bulk samples were etched to expose the grain boundaries, and their grain size was analyzed through SEM imaging. Figure 7 shows the etched microstructures with the corresponding traced binary outline masks of the grains and the overlay maps generated by ImageJ for four samples: the EOL magnet (top row), the RAS sample prepared from JM_Industrial_ powder with the addition of 1 wt.% NdH_3_ (JM_Industrial_-RAS-NdH_3_) (middle row), and the RAS and CS samples prepared with 1 wt.% NdH_3_ (JM_3X_-RAS-NdH_3_ and JM_3X_-CS-NdH_3_) (bottom row). The D_v10_, D_v50,_ and D_v90_ values obtained with ImageJ are displayed in Table 5. Unlike powder particle size analysis, where laser diffraction yields true diameters, microstructural grain size measurements reflect random two-dimensional cross-sections of grains. To approximate true grain dimensions, a section-correction factor of 1.5 was applied [12].

**Figure 8:**
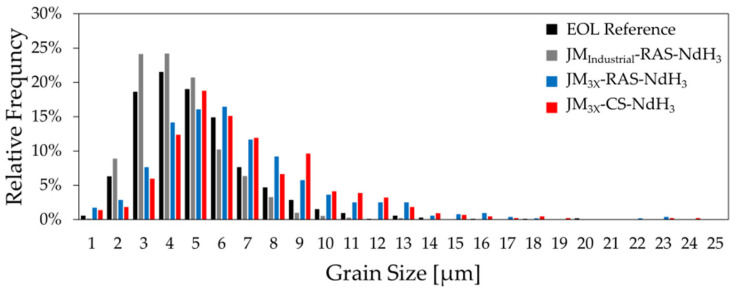
Relative frequency histograms of grain sizes (after correction) for EOL reference (n = 1046), *JM_Industrial_-RAS-NdH_3_* (n = 1496), *JM_3X_-RAS-NdH_3_* (n = 523), and *JM_3X_-CS-NdH_3_* (n = 438) samples. Grain diameters were measured from three polished and etched SEM micrographs per sample (1000x magnification) and binned in 1 µm intervals.

The contrast between the grain sizes of sintered samples prepared from the *JM_Industrial_* and *JM_3X_* powders is seen in the relative-frequency distributions in Figure 8. Here, the section-corrected Feret diameters measured by ImageJ were binned (categorized) into 1 µm intervals. The histogram highlights the differences in the grain-size distributions between the samples, revealing that the grains were smaller in the EOL reference magnet and sample *JM_Industrial_-RAS-NdH_3_* than in the RAS and CS samples prepared from powder *JM_3X_*.

The EOL magnet’s section-corrected D_v50_ grain size value of 8.2 µm (Table 5) closely matches the D_v50_ of the thrice-jet-milled *JM_3X_* powder (8.9 µm; see Table 2). This shows that jet milling using a laboratory-scale MC DecJet^®^ 50 jet mill fragmented the HPMS material via intergranular cracking, i.e., along the grain boundaries of the initial EOL magnet. In contrast, the lower D_v50_ value of 4.3 µm measured for the *JM_Industrial_* powder prepared with industrial-scale spiral jet mill LabPilot M-Jet 10 confirms that the particle size was further reduced to approximately half the grain size of the EOL magnet via intragranular fracturing. The D_v50_ value of 4.3 µm is in the range of industrially used particle sizes of 3–5 µm in the production of sintered NdFeB magnets [22].

During sintering, whether by conventional sintering or the faster RAS method, grain growth beyond the jet-milled particle size was anticipated. Prior studies have shown that sintered (RAS and CS) NdFeB magnets produced from fresh jet-milled powders exhibit grain growth factors of approximately two [12,23]. The grain size analysis of sample *JM_Industrial_-RAS-NdH_3_* revealed a D_v50_ value of ~7.3 µm, exhibiting a growth factor of 1.71, i.e., lower than previously reported. The reduced grain growth compared to fresh powders can be attributed to the high oxygen content in the *JM_Industrial_* powder (0.59 wt.%) and consequently the presence of RE oxides in the grain boundary phase, which was previously argued to reduce the mobility of the liquid fraction during liquid-phase sintering and consequently the mobility of the solid/liquid interface [24].

The D_v50_ values measured for samples *JM_3X_-RAS-NdH_3_* and *JM_3X_-CS- NdH_3_* were 10.9 and 11.2 µm, respectively, meaning that the grains grew by factors of 1.23 (RAS) and 1.26 (CS) for powder *JM_3X_*. These values are significantly lower than the growth factor reported in the literature (≈2) and the value calculated for powder *JM_Industrial_* (1.71).

A likely explanation for the reduced grain growth factor is related to the morphological specifics of powder *JM_3X_*. The intergranular cracking during jet milling preserved the shape of the EOL magnets’ grains (see Figure 3), which have previously undergone sintering followed by post-sinter annealing and are therefore in a near-equilibrated state to accommodate high density in re-sintered magnets. During liquid-phase sintering, grain growth is governed by Ostwald ripening, with the driving force originating in the chemical potential of atoms in grains of different sizes [25,26,27]. The growth driving force ∆g of a particular grain of size 2r, with r being the radius from the center of the grain to the nearest facet, is expressed in Equation (1) [28,29]:

(1) $$∆g=2\gamma V_{m}\left(\frac{1}{r*}-\frac{1}{r}\right)$$

where

γ is the solid–liquid interfacial energy;
V_m_ is the molar volume;
2r* is the critical grain size (a grain that is neither growing nor shrinking).

In a liquid-phase sintering system, as is applicable here, a range of ∆g will be found, ranging from negative values for grains smaller than the critical grain size of 2r* to positive values for grains larger than the critical grain size. Grains with positive ∆g will grow during sintering, and grains with negative ∆g will shrink. It is assumed that a fully thermodynamically equilibrated grain will have a growth driving force of ∆g = 0. Fisher and Kang [30] described the behavior of stagnant grain growth (SGG), where the critical driving force for appreciable growth ∆g_c_ is much greater than the maximum driving force for the largest grain in the system ∆g_max_. In this scenario, all grains with a positive driving force ∆g > 0 grow very slowly as their growth is controlled by the interface reaction (typically 2D nucleation), resulting in an essentially slow to stagnant growth rate. Combining the theoretical principle of SGG with the assumption of nearly thermodynamically equilibrated grains, the critical driving force is predicted to be significantly higher than the maximum driving force of the largest particle (∆g_c_ >> ∆g_max_). The nearly thermodynamically equilibrated grains would exhibit ∆g values just slightly above 0. In summary, the grain growth that occurred in the RAS and CS samples prepared from *JM_3X_* powder was stagnant and/or slowed down compared to Normal Grain Growth (NGG) due to the nearly thermodynamically equilibrated grains.

In contrast, the observed grain growth factor of the finer *JM_Industrial_* powder is in line with the NGG behavior described in [30]. The fine powder particles (D_v50_ of 4.3 µm) are morphologically rough and not faceted (see Figure 3), leading to high grain-boundary energy and a stronger driving force for grain growth (∆g > 0) during sintering.

Although finer particle sizes are often associated with enhanced intrinsic coercivity (H_ci_) due to improved domain isolation and reduced defect density [26], the present results show that this correlation is not universal. The JM_Industrial_ powder, with a smaller initial particle size (D_v50_ = 4.3 µm), did not achieve the same refinement effect as the JM_3X_ powder (D_v50_ = 8.9 µm) despite its finer particle size. Instead, the superior magnetic performance of the JM_3X_ samples is explained by the interaction between microstructural evolution and the underlying magnetic reversal mechanism.

In sintered NdFeB magnets, coercivity is thought to be primarily nucleation-controlled [24,25]. This process is highly sensitive to grain boundary quality as magnetic insulation between Nd_2_Fe_14_B grains must be maintained to ensure effective exchange decoupling. Oxide inclusions or incomplete wetting of the RE-rich phase generate weak spots with locally reduced nucleation barriers, promoting premature reversal and reducing H_ci_ [24]. The JM_3X_ particle morphology, originating from intergranular cracking and preserving near-equilibrium grain facets, facilitates stagnant grain growth (SGG) during sintering. This morphology suppresses grain coarsening (growth factor ~1.2) and helps to maintain a uniform microstructure. The combined effects of SGG and NdH_3_ addition, which increases the liquid RE-rich phase and improves magnetic insulation, eliminate easy domain-wall nucleation sites, leading to a more homogeneous reversal process. Consequently, the resulting magnets exhibit both high coercivity (up to 742 kA/m at 100 °C) and good squareness (up to 0.86).

## 4. Conclusions

This work demonstrates that Radiation-Assisted Sintering (RAS) is a viable consolidation route for recycled NdFeB powders obtained through Hydrogen Processing of Magnetic Scrap (HPMS). It enables the production of magnets with magnetic performance comparable to that achieved by conventional sintering. Combined with its previously demonstrated potential for energy-efficient sintering cycles, RAS emerges as a promising method for short-loop magnet-to-magnet recycling.

The grain size analysis of fully dense samples prepared via RAS and conventional sintering revealed that grain growth during liquid-phase sintering is strongly influenced by the initial powder morphology. When jet milling preserves the original grain shape and size of the EOL magnet, the resulting near-equilibrium powder particles exhibit stagnant growth behavior, effectively slowing grain coarsening. This shows that optimized milling conditions, which minimize intragranular fracture, can reduce grain growth during re-sintering by lowering the thermodynamic driving force.

The results also underscore the importance of minimizing additional oxygen uptake during the milling of HPMS-type materials. For an optimally milled powder with an oxygen content of 0.42 wt.%, the addition of 1 wt.% NdH_3_ fully restored—and even improved—the magnetic performance of the end-of-life (EOL) magnets. In this study, the best-performing samples achieved B_r_ = 1.18 T, (BH)_max_ = 263 kJ/m^3^, and H_ci_ = 742 kA/m (all measured at 100 °C), surpassing the original EOL magnet by approximately 4% in remanence, 9% in maximum energy product, and 6% in coercivity. The high coercivity of the samples is attributed to the combined effect of an acceptable oxidation level in the milled powder and a low grain growth factor of approximately 1.2, providing new insight for advancing short-loop magnet recycling technologies.

## Figures and Tables

**Table 1:** Chemical composition, density, and magnetic properties (measured with a permeameter at 100 °C) of the end-of-life NdFeB magnet: remanence (B_r_), intrinsic coercivity (H_ci_), maximum energy product ((BH)_max_), and the squareness factor of the demagnetization curve.

End-of-Life Magnet Characterization	
Composition [wt.%]	Nd_28.03_Fe_65.80_B_1.01_Ce_0.01_Pr_0.11_Dy_4.24_Tb_0.01_Al_0.47_Cr_0.02_Cu_0.06_Ga_0.01_Nb_0.02_Si_0.2_Sn_0.01_
Composition [at.%]	Nd_12.79_Fe_77.55_B_6.15_Ce_0.01_Pr_0.05_Dy_1.72_Tb_0.01_Al_1.15_Cr_0.03_Cu_0.06_Ga_0.01_Nb_0.01_Si_0.47_Sn_0.01_
Density [g/cm^3^]	7.45
B_r_ [T] at 100 °C	1.13
H_ci_ [kA/m] at 100 °C	699
(BH)_max_ [kJ/m^3^] at 100 °C	241
Squareness factor (H_k_/H_ci_)	0.95

**Table 2:** Oxygen content [wt.%] and laser-diffraction particle size distribution parameters D_v10_, D_v50_, and D_v90_ [µm] with calculated D_v90/10_ ratio for the *HPMS* material, powders prepared using a laboratory-scale MC DecJet^®^ 50 jet mill with consecutive milling (*JM_1X_*, *JM_2X_*, and *JM_3X_*), and a powder prepared using an industrial-scale spiral jet mill LabPilot M-Jet 10 (*JM_Industrial_*). Powders *JM_3X_* and *JM_Industrial_* were used as feedstocks for new sintered magnets.

Powder	Oxygen Content [wt.%]	D_v10_ [µm]	D_v50_ [µm]	D_v90_ [µm]	D_v90/10_ Ratio
HPMS	0.47 ± 0.06	5.6	71.9	270.7	48.2
JM_1X_	0.56 ± 0.01	2.1	8.3	19.9	9.6
JM_2X_	0.51 ± 0.00	2.0	8.0	18.2	9.0
JM_3X_	0.42 ± 0.01	4.4	8.9	15.8	3.6
JM_Industrial_	0.59 ± 0.06	2.1	4.3	7.5	3.5

**Table 3:** Densities and magnetic properties (measured at 100 °C) for the EOL magnet and bulk samples prepared from the *JM_3X_* powder with or without addition of 1 wt.% NdH_3_ by RAS and CS, followed by post-sinter annealing.

Sample	Density [g/cm^3^]	B_r_ at 100 °C [T]	H_ci_ at 100 °C [kA/m]	(BH)_max_ at 100 °C [kJ/m^3^]	Squareness Factor
EOL Reference	7.45	1.13	699	241	0.95
JM_3X_-RAS	7.58	1.17	611	238	0.68
JM_3X_-RAS-NdH_3_	7.64	1.16	742	254	0.86
JM_3X_-CS	7.62	1.17	671	254	0.78
JM_3X_-CS-NdH_3_	7.64	1.18	726	263	0.83

**Table 4:** Compositions of the phases outlined in Figure 6, quantified by EDS.

Element	Spectrum 1 [at.%]	Spectrum 2 [at.%]	Spectrum 3 [at.%]
Nd	31.4	40.2	35.5
Dy	2.9	2.8	-
Fe	2.1	2.5	5.2
O	63.6	54.5	59.3

**Table 5:** Grain size statistics (D_v10_, D_v50_, and D_v90_) for the EOL reference magnet and samples *JM_Industrial_-RAS-NdH_3_*, *JM_3X_-RAS-NdH_3_*, and *JM_3X_-CS-NdH_3_*, showing both the raw measurements from ImageJ and section-corrected values (adjusted by a factor of 1.5).

Sample	n	Measured in ImageJ			Adjusted with Factor 1.5			Growth Factor ^1^
		D_v10_ [µm]	D_v50_ [µm]	D_v90_ [µm]	D_v10_ [µm]	D_v50_ [µm]	D_v90_ [µm]	
EOL Reference	1046	3.0	5.5	9.7	4.4	8.2	14.5	-
JM_Industrial_-RAS-NdH_3_	1496	2.7	4.9	8.4	4.1	7.3	12.5	1.71
JM_3X_-RAS-NdH_3_	523	3.8	7.3	13.9	5.7	10.9	20.8	1.23
JM_3X_-CS-NdH_3_	438	4.1	7.5	14.3	6.2	11.2	21.4	1.26

^1^ The growth factor is calculated using the adjusted D_v50_ value of the measured grains in relation to the D_v50_ of the powder feedstock that was used for sintering (see Table 2).

## Data Availability

The original contributions presented in this study are included in the article. Further inquiries can be directed to the corresponding authors.

## Abbreviations

The following abbreviations are used in this manuscript:

CS	Conventional Sintering
EDS	Energy-Dispersive X-Ray Spectroscopy
EOL	End –of Life
HPMS	Hydrogen Processing or Magnet Scrap
ICP–OES	Inductively Coupled Plasma–Optical Emission Spectroscopy
JM	Jet Mill/Jet-Milled, etc.
NGG	Normal Grain Growth
RAS	Radiation-Assisted Sintering
RE	Rare Earth
SEM	Scanning Electron Microscope
SGG	Stagnant Grain Growth
